# Increased Nicotine Consumption in Australia During the First Months of the COVID-19 Pandemic

**DOI:** 10.1093/ntr/ntac275

**Published:** 2023-03-09

**Authors:** Phong K Thai, Benjamin J Tscharke, Jake O’Brien, Coral Gartner, Richard Bade, Cobus Gerber, Jason M White, Qiuda Zheng, Zhe Wang, Kevin V Thomas, Jochen F Mueller

**Affiliations:** Queensland Alliance for Environmental Health Sciences (QAEHS), The University of Queensland, 20 Cornwall Street, Woolloongabba, Queensland 4102, Australia; Queensland Alliance for Environmental Health Sciences (QAEHS), The University of Queensland, 20 Cornwall Street, Woolloongabba, Queensland 4102, Australia; Queensland Alliance for Environmental Health Sciences (QAEHS), The University of Queensland, 20 Cornwall Street, Woolloongabba, Queensland 4102, Australia; Queensland Alliance for Environmental Health Sciences (QAEHS), The University of Queensland, 20 Cornwall Street, Woolloongabba, Queensland 4102, Australia; School of Public Health, The University of Queensland, Herston, QLD, 4006, Australia; Queensland Alliance for Environmental Health Sciences (QAEHS), The University of Queensland, 20 Cornwall Street, Woolloongabba, Queensland 4102, Australia; Health and Biomedical Innovation, UniSA: Clinical and Health Sciences, University of South Australia, Adelaide 5001, South Australia, Australia; Health and Biomedical Innovation, UniSA: Clinical and Health Sciences, University of South Australia, Adelaide 5001, South Australia, Australia; Health and Biomedical Innovation, UniSA: Clinical and Health Sciences, University of South Australia, Adelaide 5001, South Australia, Australia; Queensland Alliance for Environmental Health Sciences (QAEHS), The University of Queensland, 20 Cornwall Street, Woolloongabba, Queensland 4102, Australia; Queensland Alliance for Environmental Health Sciences (QAEHS), The University of Queensland, 20 Cornwall Street, Woolloongabba, Queensland 4102, Australia; Queensland Alliance for Environmental Health Sciences (QAEHS), The University of Queensland, 20 Cornwall Street, Woolloongabba, Queensland 4102, Australia; Queensland Alliance for Environmental Health Sciences (QAEHS), The University of Queensland, 20 Cornwall Street, Woolloongabba, Queensland 4102, Australia

## Abstract

**Introduction:**

Mixed findings have been reported about the impact of the COVID-19 pandemic on smoking behavior in different populations.

**Aims and Methods:**

In this study, we aimed to quantify changes in smoking prevalence through the proxy of nicotine consumption in the Australian population from 2017 to 2020 inclusive. Estimates of nicotine consumption between 2017 and 2020 were retrieved from a national wastewater monitoring program that covers up to 50% of the Australian population. National sales data for nicotine replacement therapy (NRT) products from 2017 to 2020 were also acquired. Linear regression and pairwise comparison were conducted to identify data trends and to test differences between time periods.

**Results:**

The average consumption of nicotine in Australia decreased between 2017 and 2019 but increased in 2020. Estimated consumption in the first half of 2020 was significantly higher (~30%) than the previous period. Sales of NRT products increased gradually from 2017 to 2020 although sales in the first half of the year were consistently lower than in the second half.

**Conclusion:**

Total nicotine consumption increased in Australia during the early stage of the pandemic in 2020. Increased nicotine consumption may be due to people managing higher stress levels, such as from loneliness due to control measures, and also greater opportunities to smoke/vape while working from home and during lockdowns in the early stage of the pandemic.

**Implications:**

Tobacco and nicotine consumption have been decreasing in Australia but the COVID-19 pandemic may have temporarily disrupted this trend. In 2020, the higher impacts of lockdowns and working from home arrangements may have led to a temporary reversal of the previous downward trend in smoking during the early stage of the pandemic.

## Introduction

Smoking remains the leading preventable cause of illness and premature death globally and in Australia.^1^ Australia has been highly successful in reducing smoking prevalence as measured both by surveys^2^ and by wastewater-based epidemiology (WBE), where nicotine metabolites are used as a proxy for tobacco use^3,4^ because nicotine vaping and nicotine replacement therapy (NRT) products have been only minor contributors to total nicotine use in Australia.^5^

In early 2020, public health measures were implemented to manage the COVID-19 (or SARS-CoV-2) pandemic in Australia, including lockdowns that restricted the movement of people, public and private gatherings, and operation of businesses. Non-essential workers were expected to work from home, and school and university students were required to learn from home. These restrictions may have influenced smoking behavior.

In the last 2 years, many surveys have been conducted to understand the influence of COVID-19 on smoking and cessation behaviors around the world. They reported both an increase in quit attempts due to health concerns and increased smoking due to greater psychological stress caused by the pandemic or more opportunities to smoke due to greater time at home.^6–9^ However, survey data is less able to provide the high temporal resolution needed for assessing changes in total tobacco or nicotine consumption in the population as the COVID-19 pandemic progressed that WBE offers.^3^

This study quantified nicotine use in Australia from 2017 to 2020, covering the first year of the COVID-19 pandemic, through the proxy of mass load of cotinine and hydroxycotinine (nicotine biomarkers) in wastewater and NRT sales data.

## Method

### Wastewater Data

Estimates of total nicotine consumption were obtained from the National Wastewater Drug Monitoring Program (NWDMP) in Australia for February 2017 to October 2020, from catchments that covered up to 50% of the Australian population. For the NWDMP, daily composite influent wastewater samples were collected from each participating wastewater treatment plant (WWTP) for up to 7 consecutive days on a bimonthly (capital cities) and quarterly (regional places) basis. Biomarkers of nicotine use, cotinine, and hydroxycotinine, were analyzed in all samples to estimate consumption by the WWTP catchment population. Detailed information about the sampling campaigns of the NWDMP are available elsewhere.^10^

The per capita daily estimated consumption of nicotine from individual WWTPs were averaged over each semi-annual period to generate the mean and standard deviation values for that period.

### Nicotine Sales Data

Monthly data of wholesale Australian pharmacy sales of NRT products for the period of 2017–2020 were provided by IQVIA (iqvia.com). The IQVIA claim the data captures about 96% of all over-the-counter and prescription pharmaceutical sales to pharmacies in Australia. Wholesale supply of NRT to general retailers (supermarkets and convenience stores), and hospitals are not included. Total nicotine was determined by summing the mass across all nicotine products by half year and then converted to the per-capita daily consumption of nicotine through NRT.

### Statistical Analysis

Descriptive statistics including the arithmetic means, standard deviation, and medians were generated for the semi-annual national per capita mass load of nicotine biomarkers in wastewater across the whole dataset. Univariate analysis was performed in SPSS 22 using a general linear model to compare the changes of slopes between every two-year period.

## Results

### National Trend of Nicotine Use in Australia 2017–2019

**Figure 1.**
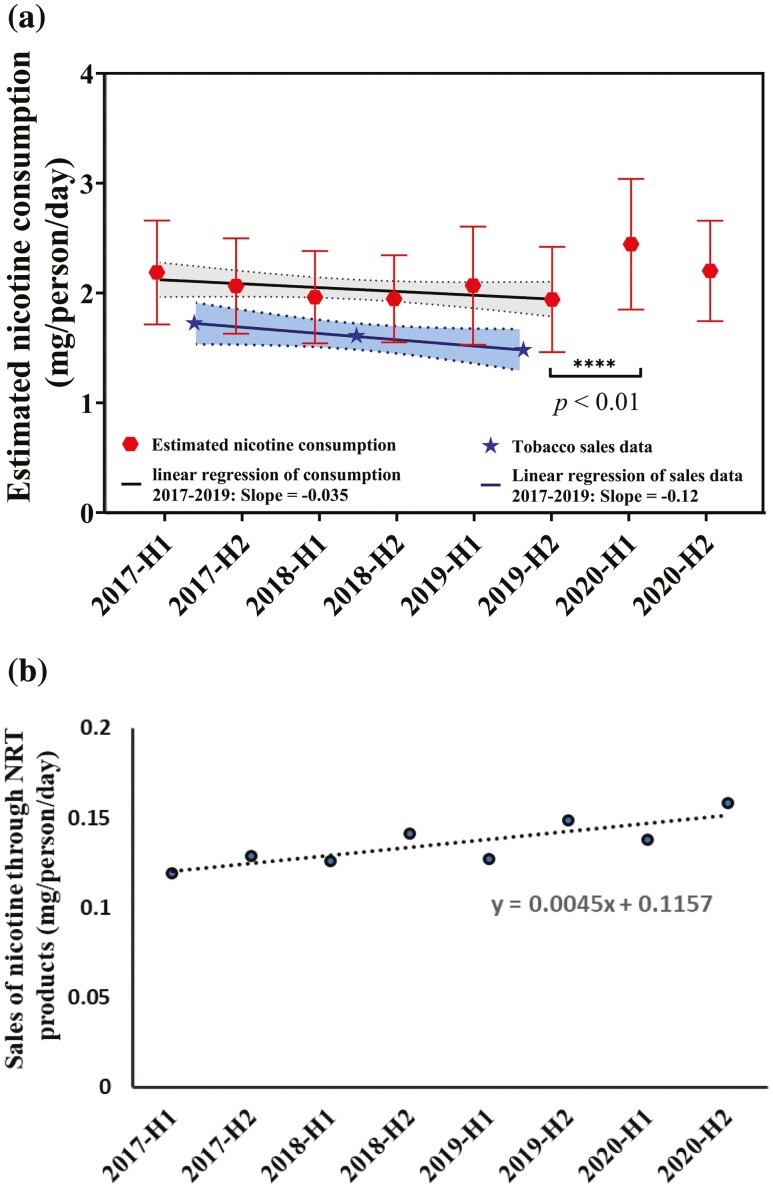
(a) Total estimated per capita nicotine use by wastewater (2017–2020; H1 = first half year) and by tobacco sales data (2017–2019), 95% confidence intervals were showed for the linear regression lines of both data set while standard error bars were showed for estimated nicotine consumptions; and (b) sales of nicotine replacement therapies 2017–2020.

Semi-annual estimates from the NWDMP data indicated a ~5% annual decrease in total nicotine use from 2017 to 2019 (Figure 1a). Consistent with increased quitting activity during this time, pharmacy-based sales of NRT increased ~5% over the same period (Figure 1b). Assuming that NRT sales correspond to NRT use, the products currently contribute < 10% of the total nicotine consumption in Australia.

### Nicotine use in Australia in 2020

The estimated total nicotine consumption in the first half of 2020 was significantly higher than estimated for the second half of 2019 (Figure 1a), with the average daily mass load increasing from 1.4 to 1.8 mg/person/day (~30%). Estimated consumption decreased in the second half of 2020 (1.6 mg/person/day), although the change from 2019 was not significant (*p* > .05). Despite this higher level of overall nicotine use, NRT sales continued to increase through 2020, suggesting NRT-assisted quitting activity continued to increase through 2020 despite an increase in overall nicotine use. Interestingly, NRT sales are consistently lower in the first half of the year than in the second half (Figure 1b).

## Discussion

Our estimates until the end of 2019 are consistent with the findings of our long term study in an Australian population from 2012 to 2017.^3^ Smoking prevalence has decreased steadily in Australia, as reported for the period 2001–2017 by national survey data.^2^ As tobacco cigarettes are the most used nicotine product in Australia, the reduction in smoking has contributed considerably to the decrease in total mass of nicotine used by the Australian populations as estimated by WBE studies up to 2019.^3^ Although there was an increase in consumption of NRT such as nicotine patches (Figure 1b), consistent with the decreasing smoking prevalence, NRT only accounts for a small portion of total nicotine consumption, although this proportion may increase with increasing use of quit assistance and decreasing smoking prevalence.

In March 2020, Australia had the first wave of COVID-19 cases. The federal government quickly imposed measures to reduce transmission risks through actions such as lockdowns, social-distancing restrictions, and work-from-home requirements. Those actions and the uncertainty about the danger of COVID-19 posed significant stress on the population. In the second half of 2020, many of the restrictions were eased and restrictions were implemented at a local level by State governments in response to local outbreaks.

The uncertainties during the early months of the COVID-19 pandemic may have had a detrimental impact on quitting activity. The social and economic changes, such as movement restrictions and job losses, caused by the COVID-19 pandemic, have increased mental health issues, which may have decreased motivation to quit smoking or quit self-efficacy, and/or increased relapse to smoking among people who recently quit.^6^ Furthermore, more time spent at home rather than in workplaces or other settings may have increased opportunities to smoke, leading to heavier smoking. In the US, tobacco sales reportedly increased during the pandemic.^7^ In Italy, an increase in cigarette smoking was associated with an increase in mental distress during lockdowns.^8^ In other countries, such as the UK and the Netherlands, the number of people who smoked more during the pandemic was offset by those who smoked less.^6,11^

In Australia, early in 2020, there was an increase in downloads of the government’s My Quit Buddy app, suggesting an increase in quitting activity.^12^ The economic impacts of the pandemic may have been a motivation to quit smoking. Australia has very high cigarette prices due to a series of annual tobacco tax increases of 12.5% applied from 2013 to 2020. During this time, the high cost of smoking overtook health reasons as the most commonly cited reason for quitting or cutting down.^13^ However, health reasons to quit may also have been particularly salient in early 2020 due to media reports that smoking may increase the risk of severe COVID-19 outcomes.^14^ A cohort study of Australian adults found that smoking (of any frequency) slightly decreased during the pandemic due to health concerns.^15^ However, at the same time, a survey by the National Heart Foundation reported that one third of respondents increased their smoking while another third reduced their smoking for various reasons, for example, health concerns, stress, employment situation, free time,^16^ similar to the findings of annual survey of people who use drugs across Australia^17^ as well as a UK study.^11^

Our results suggest a significant increase in nicotine use in Australia early in the pandemic (first half of 2020) when many restrictions were widely enforced across the country. As restrictions were eased, nicotine use decreased again although it was still higher in late 2020 than pre-pandemic.

Although we lack tobacco sales data for 2020 to corroborate our observed increase, estimates from wastewater analysis fit well with available tobacco sales data for the 2017–2019 period, reported annually by the tobacco industry as shown in Figure 1a,^18^ after accounting for the increase in sales of NRT products over the same period. Additionally, a significant decrease in the number of people seeking help to quit smoking has been reported during the second lockdown in the State of Victoria in September/October 2020.^19^ Our data fit with increased smoking at this time, similar to reports from the US, as attention was drawn away from quitting efforts to other issues caused by the pandemic.^7^ Fewer people quitting smoking and some increasing the amount they smoked, as reported by the Heart Foundation survey, is likely to have contributed to the increased overall nicotine use early in the pandemic. As smoking can cause higher morbidity and mortality risks than COVID-19 in Australia, it is important to maintain progress in reducing smoking by supporting quit efforts.

Our aggregate data of NRT products shows a stable increase in sales, which continued during 2020. This could be contradictory to the explanation that reductions in quitting resulted in the increase in nicotine use seen in 2020. However, it is also possible that there was a reduction in quitting but that this occurred only for unassisted quits and hence did not impact on NRT sales trends. An alternative explanation is that the main cause of increase in nicotine use observed in 2020 was increased intensity of smoking or increased illegal vaping of nicotine rather than reduced quitting.

Although there are no sales data available for nicotine vaping products because it was illegal during that time, survey data suggested that nicotine vaping remains a minor contributor to total nicotine use compared to smoking.^5^ Other limitations of our study include the lack of sales data for NRT purchased over-the-counter in supermarkets or other general retailers, and administered in hospitals.

## Data Availability

The data underlying this article will be shared on reasonable request to the corresponding author.
